# Experiences applying for and understanding health insurance under Massachusetts health care reform

**DOI:** 10.1186/s12939-016-0397-6

**Published:** 2016-07-18

**Authors:** Rachel Nardin, Leah Zallman, Assaad Sayah, Danny McCormick

**Affiliations:** Cambridge Health Alliance, 1493 Cambridge St; Macht 420, Cambridge, MA 02139 USA; Institute for Community Health, Malden, MA USA; Harvard Medical School, Boston, MA USA

**Keywords:** Massachusetts, Health reform, Insurance, Experiences

## Abstract

**Background:**

The Affordable Care Act was modeled on the Massachusetts Health Reform of 2006, which reduced the number of uninsured largely through a Medicaid expansion and the provision of publicly subsidized insurance obtained through a Health Benefits Exchange.

**Methods:**

We surveyed a convenience sample of 780 patients seeking care in a safety-net system who obtained Medicaid or publicly subsidized insurance after the Massachusetts reform, as well as a group of employed patients with private insurance.

**Results:**

We found that although most patients with Medicaid or publicly subsidized exchange-based plans were able to obtain assistance with applying for and choosing an insurance plan, substantial proportions of respondents experienced difficulties with the application process and with understanding coverage and cost features of plans.

**Conclusions:**

Under the Affordable Care Act, efforts to simplify the application process and reduce the complexity of plans may be warranted, particularly for vulnerable patient populations cared for by the medical safety net.

## Background

The United States has been anomalous among developed countries in lacking a system of universal healthcare coverage. Financial barriers to care, particularly for low income and uninsured people and racial and ethnic minorities, have been considerably higher in the US than in other wealthy nations [1, 2]. In order to address deep inequalities in access to care and health, the US implemented the landmark Patient Protection and Affordable Care Act (ACA) in 2014. The ACA is a nationwide policy intervention designed to expand access to medical care through the largest increase in insurance to low income people in US history; once fully implemented it is expected to cover 30 million of the approximately 50 million American that were uninsured prior to this reform [3]. The new forms of insurance provided under the ACA, however, must be applied for through newly created health exchanges and kept active by periodically providing proof of continued eligibility [4, 5]. In addition, in order to have these new insurances pay for needed medical services, recipients must be able to understand and be able to effectively utilize these insurances. The extent to which the ACA will ultimately be able to reduce barriers to accessing care will depend critically on the experiences of Americans in obtaining, keeping and using new insurance provided under the ACA.

Under the Affordable Care Act (ACA), non-elderly American adults began accessing new health insurance options in October 2013. This expansion is occurring by extending Medicaid coverage (comprehensive government insurance for low income individuals) to additional low-income residents in the 28 states that have agreed to a Medicaid expansion and by offering private health insurance plans to consumers through state Health Benefits Exchanges. This private insurance is federally subsidized via premium tax credits for those with incomes between 100 % and 138 % of the federal poverty level (depending on whether states expanded Medicaid) and 400 % of the federal poverty level [4].

States are allowed to run their own exchanges, use the federal exchange or build an exchange in partnership with the federal government. All states must also implement streamlined Medicaid enrollment processes that offer individuals multiple ways to apply (online, by phone, in person, by mail), rely on electronic data to verify information and attempt to provide real time eligibility determinations. These processes must be coordinated across Medicaid and the Benefits Exchange to create a “no wrong door” enrollment system.

If consumers, because of inadequate information have difficulty understanding the application process or procedures for maintaining coverage (such as the periodic redetermination of plan eligibility), some may be deterred from obtaining or keeping their insurance. Similarly, if consumers’ lack of understanding leads some to select plans that have either unaffordable premiums or co-payments, or lack of coverage for needed services, coverage may be lost or needed services forgone.

There is significant evidence that non-elderly Americans have difficulty understanding basic health insurance terms and how insurance works [6–8]. The majority of the newly insured under the ACA will obtain either Medicaid or publicly subsidized private insurance through a Health Benefit Exchange [9]. Research has shown that low-income populations, especially those with low numeracy and literacy skills, as well as the previously uninsured, have particular difficulty comprehending health plan information [10–13]. This raises concern that some consumers may have difficulty accessing, retaining and using insurance under the ACA, but because the ACA rollout is so new, there is only scant data on consumer experiences under the reform [14].

The ACA was modeled largely on the Massachusetts reform of 2006, which also aimed to provide universal coverage through a Medicaid expansion and public subsidy of private insurance for low-income residents, called Commonwealth Care (CWC). Massachusetts created a Health Benefits Exchange, called the Health Connector, consistent with the requirements for an exchange subsequently mandated by the ACA. The state created a single, seamless paper Medical Benefit Request and online Virtual Gateway through which low income individuals could apply for publicly subsidized insurance. The state’s Office of Medicaid then made a determination of eligibility for either Medicaid or CWC based on information provided regarding income and immigration status. Individuals deemed eligible for Medicaid were provided with their choice of a primary care physician (PCC) or managed care option (MCO) Medicaid plan. Those deemed eligible for CWC were assigned, based on income, to one of three types of CWC, which differed by whether a premium payment was required and in their cost-sharing features. Residents chose a CWC plan from among those offered by the five insurance companies approved by the state to offer subsidized plans through the exchange, either by calling the CWC customer service line or via the Health Connector website; any required premiums were paid through the Health Connector. The Health Connector also offered non-subsidized private insurance plans for higher income individuals and small businesses.

Under the Massachusetts reform, 84 % of newly insured residents received Medicaid or CWC, with only 17 % obtaining insurance through an employer or obtaining non-subsidized insurance through the exchange [15]. There has been one study of the small fraction of Massachusetts residents using the Health Connector to purchase non-subsidized plans, showing that 40 % found plan information difficult to understand and 20 % wished they had help narrowing plan choices [16]. To our knowledge, however, there is no published data on experience choosing and using health insurance plans among those obtaining Medicaid or publicly subsidized insurance under the Massachusetts reform.

Most developed countries and many developing countries approach the ethical imperative to strive for health care equity by utilizing universal health care coverage systems [17]. Under such systems, because they apply universally, rules governing eligibility for and procedures for obtaining, keeping and utilizing coverage are relatively simple in comparison with the multiplicity and variety of private and public insurance plans in the US [18, 19]. In order to improve health inequalities, the ACA and the MA reform created a new set of public and private coverage types that potentially add additional complexity and could be challenging to acquire and use, particularly for people with low literacy and health literacy levels or who have limited English language proficiency.

We sought to study the experience of Massachusetts residents in obtaining, understanding and using health insurance after the creation of a Health Benefit Exchange to shed light on what might ultimately be expected under the more recently implemented ACA, particularly among vulnerable patient populations that were the primary target of both reform laws and among those with actual experience seeking care. Such an analysis could also hold insights for other countries that might consider moving toward a mixed healthcare financing model built on employer based private insurance with public and publicly subsidized private insurance for the poor as the US has adopted under the ACA [4] and Massachusetts did under its reform [20].

## Methods

### Study design and setting

We conducted face to face interviews with a convenience sample of patients presenting to any of three Emergency Departments of the state’s second largest safety net hospital system, located in three communities in eastern Massachusetts (Everett, Somerville and Cambridge, MA), between August 2013 and January 2014, as previously described [21]. The Cambridge Health Alliance Institutional Review Board approved the study protocol.

For the present study, we included the 780 patients enrolled in a larger study [21] who had obtained Medicaid or CWC since the start of the Massachusetts reform, as well as a group of non-self-employed, working patients with private (commercial) insurance, most of whom were likely to have employer-based private insurance. Insurance status and type were determined by electronic querying of a continuously updated insurance database maintained by a consortium of all Massachusetts health insurers, including public payers [22]. This database allows real-time determination of insurance type and status with nearly 100 % accuracy. We recorded patients as having Medicaid if they were covered by any one of the seven subtypes of Medicaid available in Massachusetts. Patients with more than one type of insurance were excluded to allow us to isolate the impact of each insurance type.

### Study subjects

We included patients aged 18–64 years who spoke one of the four most commonly spoken languages in the geographic area (English, Spanish, Portuguese, or Haitian Creole) and with an Emergency Severity Index (ESI) of 2–5 (excluding the most severely ill, ESI of 1). This score is a validated emergency department triage algorithm that stratifies patients into five groups from 1 (most urgent) to 5 (least urgent) [23]. We excluded patients with altered mental status, the inability to speak, those who had learned of a change in insurance on the day of the interview and those who reported enrolling in Medicaid prior to the rollout of the Massachusetts reform in July 2006.

### Study recruitment and data collection

Trained research assistants stationed in the Emergency Department reviewed the demographic and insurance information of all patients presenting for care. For patients meeting study entry criteria, the research assistant approached the patient to invite participation, obtain informed consent and verbally administer the survey. For patients whose primary language was Spanish, Portuguese or Haitian Creole, an interpreter or bilingual research assistant was used for study consent and survey administration. Participants were offered a $10 gift card as compensation for their time. All interviews were conducted between 9:00 am and 11:00 pm on all days of the week.

### Survey development

Details of the development and pilot testing of the survey instrument have been previously described [21]. Briefly, we developed a survey instrument to assess the experience of applying for insurance, sources of information used to choose an insurance plan, knowledge about plan features and effect of knowledge on the use of health care. We assessed knowledge of co-pay amounts for those respondents with publicly subsidized insurance, where co-pays could be accurately confirmed from published sources [24, 25]. Trained medical interpreters translated the survey into Spanish, Portuguese and Haitian Creole.

### Statistical analysis

The outcomes of interest were the multiple measures of patient experience in applying for, understanding and using health insurance. For each outcome we calculated the percentage of respondents’ answers overall and according to insurance type and compared these using chi-square tests. In order to assess potential non-response bias, we compared the gender, mean age, insurance type and distribution of Emergency Severity Index scores between respondents and non-respondents using the Student’s t-test and chi-square tests respectively. All analyses were performed using SAS software version 9.3 (SAS Institute, Cary, North Carolina).

## Results

There were 780 study participants; 212 people out of 992 (21 %) declined to participate. Non-responders were significantly more likely to be acutely ill with an ESI of 2 or 3 than responders (64 % vs. 54 %, *p* = 0.0124), but there were no differences in gender, age or insurance type. Of the 780 participants, 19 % had CWC, 50 % had Medicaid and 31 % were non self-employed with private (commercial) insurance.

Table 1 shows the demographics of the sample. The study sample included significant numbers of subjects from vulnerable populations, including the poor, members of racial or ethnic minorities and the unemployed. Those with publicly subsidized insurance were more likely to be female, foreign born, non-white, low income and were less likely to have at least a high school education and be employed than the privately insured. They were also slightly more likely than the privately insured to report less than very good health status and to have filled a prescription or been hospitalized in the past year, although not to have seen a doctor in the past year.

**Table 1:** Demographics of Sample

	Insurance Type					Group *p*- value
	Total *N* = 976	Medicaid *N* = 517 (53 %)	CWC1 *N* = 97 (9.9 %)	CWC2/3 *N* = 52 (5.3 %)	Private *N* = 310 (31.8 %)	
Male	348 (38.5 %)	155 (32.8 %)	33 (36.7 %)	15 (29.4 %)	145 (50 %)	<0.0001
Foreign-born	566 (62.6 %)	284 (60 %)	56 (62.2 %)	16 (31.4 %)	210 (72.4 %)	<0.0001
Education ≥ high school	778 (86.6 %)	378 (80.8 %)	79 (87.8 %)	44 (86.3 %)	277 (95.9 %)	<0.0001
Race						<0.0001
Black, non-Hispanic	141 (16 %)	74 (16.2 %)	19 (22.4 %)	9 (18 %)	39 (14 %)	
White, non-Hispanic	431 (49.5 %)	199 (43.5 %)	46 (54.1 %)	15 (30 %)	171 (61.5 %)	
Hispanic	245 (28.1 %)	156 (34.1 %)	15 (17.7 %)	23 (46 %)	51 (18.4 %)	
Other	54 (6.2 %)	29 (6.3 %)	5 (5.9 %)	3 (6 %)	17 (6.1 %)	
Age						0.0047
18–30	338 (37.5 %)	173 (36.7 %)	40 (44.4 %)	10 (19.6 %)	115 (39.8 %)	
31–45	325 (36 %)	177 (37.5 %)	26 (28.9 %)	16 (31.4 %)	106 (36.7 %)	
46–65	239 (26.5 %)	122 (25.9 %)	24 (26.7 %)	25 (49 %)	68 (23.5 %)	
Annual Income						<0.0001
< $20,000	527 (58.3 %)	367 (77.6 %)	59 (65.6 %)	24 (47.1 %)	77 (26.6 %)	
≥ $20,000	377 (41.7 %)	106 (22.4 %)	31 (34.4 %)	27 (52.9 %)	213 (73.5 %)	
Employed	566 (62.7 %)	206 (43.6 %)	56 (62.2 %)	43 (84.3 %)	261 (90.3 %)	<0.0001
ESI						0.1917
2 or 3	488 (54.5 %)	270 (57.5 %)	40 (46 %)	26 (53 %)	152 (52.6 %)	
4 or 5	408 (45.5 %)	200 (42.6 %)	47 (54 %)	24 (48 %)	137 (47.4 %)	
Any Rx since on plan	685 (76.1 %)	381 (80.9 %)	68 (75.6 %)	40 (78.4 %)	196 (68.1 %)	0.0010
Average monthly rx since on plan						0.0006
1–2	214 (46.6 %)	102 (39.4 %)	23 (44.2 %)	14 (51.9 %)	75 (62 %)	
≥ 3	245 (53.4 %)	157 (60.6 %)	29 (55.8 %)	13 (48.2 %)	46 (38 %)	
Doctor visit in past year						0.0013
0	173 (19.3 %)	79 (16.8 %)	18 (20.2 %)	7 (14.3 %)	69 (23.8 %)	
1–2	343 (38.2 %)	167 (35.5 %)	27 (30.3 %)	21 (42.9 %)	128 (44.1 %)	
≥ 3	382 (42.5 %)	224 (47.7 %)	44 (49.4 %)	21 (42.9 %)	93 (32.1 %)	
Hospitalization past year	208 (23.3 %)	129 (27.7 %)	25 (28.1 %)	9 (17.7 %)	45 (15.6 %)	0.0009
Reported Health Status						<0.0001
Excellent or very good	316 (39 %)	125 (30.5 %)	25 (31.3 %)	22 (51.2 %)	144 (52 %)	
Good or fair	494 (61 %)	285 (69.5 %)	55 (68.8 %)	21 (48.8 %)	133 (48 %)	

Table 2 shows respondents’ experiences applying for insurance. Of those who reported they had played a role in applying for insurance, 24 % found it difficult to figure out how to apply for their current insurance and 28 % found it difficult to complete the application process; respondents with Medicaid and CWC found it more difficult than those with private insurance. Overall, 89 % reported having information about health plan choices available in their primary language; this was significantly lower for those with CWC than with Medicaid or private insurance.

**Table 2:** Consumer Understanding of Current Health Insurance

						Group *p*-value
	Total *N* = 976	Medicaid *N* = 517	CWC1 *N* = 97	CWC2/3 *N* = 52	Private *N* = 310	
Do not understand coverage and costs^a^	203 (22.2 %)	95 (19.9 %)	15 (16.7 %)	12 (23.5 %)	81 (27.6 %)	0.046
Not confident in knowledge of coverage and costs^b^	310 (33.8 %)	140 (29.1 %)	28 (30.8 %)	21 (40.4 %)	121 (41.2 %)	0.004
Uncertainty caused delay or avoidance of medical or mental health care^c^	97 (32.1 %)	46 (34.1 %)	9 (32.1 %)	6 (28.6 %)	36 (30.5 %)	0.6325
Publicly-insured respondents reporting a copay for PCP visit or medication	*N* = 666	*N* = 517	*N* = 97	*N* = 52	n/a	
Correctly stated copay for PCP visit	572 (85.9 %)	465 (89.9 %)	82 (84.5 %)	25 (48.1 %)		<0.0001
Correctly stated copay for medication	366 (55.0 %)	290 (56.1 %)	56 (57.7 %)	20 (38.5 %)		0.043

^a^missing data for 69 subjects

^b^missing data for 64 subjects

^c^missing data for 8 subjects

Of those who had a role in applying for health insurance, 25 % tried to get information on plan choices through a state telephone help line, 32 % by speaking with someone at a health center, hospital or community based organization such as a shelter, halfway house, prison or school, and 29 % by using a plan or the Health Connector website. Those who sought assistance from any of these sources found it helpful (77 % found a telephone line helpful, 96 % found a person helpful and 76 % found a website helpful); this did not differ by insurance type.

Overall, 41 % of respondents reported that since signing up for their current plan they had had to submit additional information or paperwork to keep insurance active; this was significantly more likely for those with publicly subsidized insurance than with private insurance. Regardless of insurance type, 35 % agreed that it was difficult to submit the paperwork needed in time to prevent insurance from being cancelled.

Table 3 shows respondents’ experience choosing an insurance plan. Of the 305 respondents who played a role in choosing their insurance plan, 89 % agreed that at the time they signed up for insurance they were able to get questions about the plans answered and 87 % of respondents had all of the information about health plans needed to make a good decision about which plan to choose; respondents with CWC were the least likely to feel that they had information. Eighty percent had all of the information about costs needed to make a good decision, yet 38 % of respondents said the information provided regarding plans was hard to understand; 37 % agreed it would have been easier to choose a plan if there were fewer plans to choose from. None of these measures differed significantly by insurance type.

**Table 3:** Consumer Experience Applying for Health Insurance

	Insurance Type					Group *p*- value
	Total	Medicaid	CWC 1	CWC 2/3	Private	
Had a role in applying for health insurance	530 (56.4 %)	319 (64.4 %)	58 (61.7 %)	31 (59.6 %)	122 (40.8 %)	<0.001
Difficult to figure out how to apply for insurance	128 (24.2 %)	82 (26.0 %)	21 (35.6 %)	11 (35.5 %)	14 (11.4 %)	<0.0005
Difficult to complete application process	156 (29.9 %)	103 (33.1 %)	25 (42.4 %)	12 (38.7 %)	16 (13.2 %)	<0.0001
Did not have information about health plans in primary language	57 (10.9 %)	32 (10.1 %)	11 (19.3 %)	6 (19.4 %)	8 (6.7 %)	0.031
Tried to get plan information through a state telephone help line	78 (24.2 %)	38 (22 %)	18 (52.9 %)	13 (59.1 %)	9 (9.6 %)	<0.0001
Information obtained from telephone helpline was somewhat or very helpful	168 (78.9 %)	116 (81.1 %)	25 (73.5 %)	17 (81.0 %)	10 (66.7 %)	0.4916
Tried to get plan information by speaking with someone at a health center, hospital or community based organization	175 (19.4 %)	127 (26.9 %)	20 (22.2 %)	10 (19.6 %)	18 (6.2 %)	<0.0001
Information obtained from person was somewhat or very helpful	168 (95.5 %)	123 (96.1 %)	20 (100 %)	10 (100 %)	15 (83.3 %)	0.0540
Tried to get plan information through a plan or Health Connector website	121 (26.8 %)	53 (19.4 %)	20 (40.8 %)	13 (46.4 %)	35 (34.7 %)	<0.0001
Information obtained from website was somewhat or very helpful	96 (77.4 %)	41 (75.9 %)	17 (85.0 %)	9 (69.2 %)	29 (78.4 %)	0.7424
Had to submit additional paperwork to keep insurance active	342 (42 %)	250 (57.9 %)	45 (59.2 %)	25 (58.1 %)	22 (8.4 %)	<0.0001
Difficult to submit the paperwork needed to keep insurance active	112 (33.5 %)	77 (31.4 %)	19 (40.9 %)	7 (30.4 %)	10 (45.5 %)	0.382

Table 4 shows respondents’ understanding of their current insurance plan, overall and by insurance type. Overall, 24 % of respondents reported not understanding the benefits and costs of their coverage, and 35 % reported that they did not feel confident in their knowledge of this information. Confidence varied significantly by insurance type, with those having private insurance reporting less confidence in their knowledge. Among respondents who were not confident, 27 % delayed or avoided medical or mental health care due to this uncertainty; this did not vary by insurance type. Among those with publicly subsidized insurance, 53 % correctly stated their co-pay for medication and 83 % correctly stated their co-pay for a primary care doctor visit; those with Medicaid were significantly more likely to correctly state their co-pay amount for a doctor visit than those with CWC.

**Table 4:** Consumer Experience Choosing an Insurance Plan

	Insurance Type					Group *p*- value
Had a role in choosing health insurance company or plan	Total *N* = 360	Medicaid *N* = 169	CWC 1 *N* = 49	CWC 2/3 *N* = 30	Private *N* = 112	
Had all information needed to choose a plan wisely	308 (88 %)	147 (90.2 %)	38 (79.2 %)	21 (77.8 %)	102 (91.1 %)	0.469
Found it hard to understand information about plans	132 (38.0 %)	59 (37.1 %)	17 (34.7 %)	17 (56.7 %)	39 (35.8 %)	0.176
Agreed it would have been easier to choose a plan with fewer plans to choose from	98 (39.4 %)	44 (43.6 %)	11 (37.9 %)	8 (38.1 %)	35 (35.7 %)	0.721
Agreed had sufficient information about costs to choose plan wisely	285 (81.4 %)	134 (82.2 %)	34 (75.6 %)	22 (73.3 %)	95 (84.8 %)	0.355
Were able to get questions about plans answered	306 (88.7 %)	140 (87.0 %)	40 (85.1 %)	25 (89.3 %)	101 (92.7 %)	0.422

## Discussion

Under the ACA, an estimated 20 million people have gained coverage or enrolled in a new plan since October 2013. The majority has gained this new coverage either through Medicaid (6 million with Medicaid or Children’s Health Insurance Plan) or by selecting a plan through the state Health Benefit Exchanges (8 million) [9]. We studied the experience of a convenience sample of Massachusetts residents obtaining insurance after the creation of a streamlined enrollment process and Health Benefit Exchange under the Massachusetts reform of 2006. We found that substantial proportions of those participating in this process experienced significant challenges acquiring, keeping and using new insurance; this may offer lessons for improvement under the ACA.

Our study sample, drawn from patients receiving care in any of three Emergency Departments of a large safety net provider, had substantial representation from vulnerable patient populations- racial and ethnic minorities, the unemployed, those with poor health and high health care utilization and low incomes-for whom the Massachusetts reform and the ACA were principally designed. In this policy-relevant population, we found that about a third of low-income individuals still had difficulty applying for insurance, despite the creation of a single seamless process for accessing publicly subsidized health insurance as part of Massachusetts’ health care reform. In contrast, far fewer of those with employer-based private insurance experienced such difficulties. This raises the possibility that the complexity of the application process placed a disproportionate burden on low-income people. Demographic and financial information had to be provided to allow eligibility determination; applicants were then assigned to a Medicaid plan or informed that they qualified for a CWC plan, the latter of which they then selected by web, phone or mail. Although we did not collect detailed information on the application process experienced by those with employer- based private insurance, many employer-based plans in the US enroll employees at the start of employment with automatic re-enrollment afterwards, a process requiring less consumer input than that required to apply for means-tested public insurance. Our finding that the privately insured were much less likely than the publicly insured to have to submit additional paperwork to keep insurance active is consistent with this hypothesis.

Massachusetts put significant energy and resources into outreach and education to facilitate consumer enrollment in insurance plans [26]. The state operated a telephone help line. Community health centers, hospitals and other community-based organizations received grants to provide in-person help with the application process. Our study supports that these attempts to provide information to low-income consumers were largely effective, with the vast majority of those who chose a plan reporting they were able to get their questions about plans answered. At least three-quarters of respondents who sought information from these sources were likely to find it helpful.

However, substantial minorities of low-income consumers perceived that they did not have the information they needed to choose a publicly subsidized plan wisely. This was especially true for those selecting a CWC plan through the state Health Benefit Exchange. This was better for those accessing Medicaid, where there was little choice involved for consumers. This points to significant deficiencies in the ability of the MA reform implementation to provide adequate insurance information to meet the needs of consumers.

About 20 % of respondents with publicly subsidized insurance reported they did not understand the coverage and cost features of their plan and about a third were not confident in their knowledge of coverage and costs. This lack of confidence was not misplaced, as only about half could correctly state their co-pay for medication and only approximately one quarter could correctly state their co-pay for a primary care doctor visit. We did not find that difficulties understanding plan features were significantly worse for those with publicly-subsidized insurance than those with employer-based private coverage; in fact, more respondents with private coverage reported a lack of confidence in their knowledge of plan coverage and costs. This is consistent with prior literature demonstrating poor understanding among consumers of basic health insurance terms and how insurance works [6–8].

Furthermore, our finding that over a quarter of respondents who lacked confidence in their knowledge about their insurance plan delayed or avoided medical or mental health care, emphasizes that poor understanding of insurance benefits and costs may adversely affect utilization of health care services, potentially resulting in avoidance of needed care.

The major limitation of our study is that the sample is drawn from the Emergency Departments of safety net hospitals in three communities in eastern Massachusetts, where patients are more likely than the general population to have lower health literacy rates and limited English proficiency. Massachusetts residents as a whole are largely white (82 %) and have high educational levels (40 % have college degrees) and higher incomes (11.6 % in poverty) relative to most US states [27]. The study was designed to gain in-depth understanding in a convenience sample of patients seeking and needing emergency care at safety net hospitals and was not intended to be representative of the general population in MA. Our results cannot be generalized to the state as a whole nor to residents who do not require urgent care. However, patients with publicly subsidized forms of insurance are more likely to seek care in safety net hospitals so this research design allowed us to identify a population of patients with high policy relevance: those with publicly subsidized forms of insurance with representation from vulnerable patient groups that were the target of health care reform. Also, by recruiting respondents from the Emergency Department, we were able to verify with 100 % accuracy the insurance status and type, thus reducing the high rates of error introduced by asking respondents to identify their insurance type [28]. This is essential in ascertaining how our outcomes differed by public insurance type.

Our sampling frame also resulted in a substantially higher response rate than population-based surveys, decreasing the chance of non-response bias. We were not able to collect detailed information on the plan structure of our respondents with private insurance, which limited our understanding of the details of consumer-reported difficulties with these plans.

## Conclusions

Although the Massachusetts health reform expanded coverage significantly, our study of a population of Massachusetts residents seeking emergency care at a safety net hospital system suggests that many low-income residents had difficulties in applying for and understanding health insurance plans after the creation of a single, streamlined process for accessing publicly subsidized insurance. Our findings suggest that under the ACA, continued efforts to simplify the insurance application process and insurance features are will be needed and assistance throughout the enrollment continuum, including re-enrollment, is critical. As coverage continues to expand under the ACA, we must recognize that the provision of multiple plans with variable coverage and cost features and a continued reliance on means-tested publicly subsidized insurance will result in continued difficulty for some in accessing, understanding and using health insurance. Our findings could provide insight into reforms of healthcare insurance provision built on a mix of private and public funding and individual mandates that both wealthy and less wealthy countries may contemplate implementing in the future.

## Abbreviations

ACA, Affordable care act; CWC, Commonwealth Care insurance plan; ESI, Emergency Severity Index; MCO, managed care option Medicaid plan; PCC, primary care physician Medicaid plan.
